# Dedifferentiated liposarcoma with a rare presentation of disseminated intraperitoneal sarcomatosis: A case report

**DOI:** 10.1016/j.ijscr.2019.06.051

**Published:** 2019-06-26

**Authors:** Mingzhe Cai, Caroline Ching Hisa Siew, Timothy Kwang Yong Tay, Grace Hwei Ching Tan

**Affiliations:** a Department of General Surgery, Singapore General Hospital, 20 College Road, 169856, Singapore; b Department of General Surgery, Tan Tock Seng Hospital, 11 Jln Tan Tock Seng, 308433, Singapore; c Department of Anatomical Pathology, Singapore General Hospital, 20 College Road, 169856, Singapore; d Division of Surgical Oncology, National Cancer Centre Singapore, 11 Hospital Dr, 169610, Singapore

**Keywords:** Liposarcoma, Dedifferentiated liposarcoma, Intraperitoneal sarcomatosis, MDM2

## Abstract

**Introduction:**

Dedifferentiated liposarcoma is rare and tends to present with discrete tumors in the retroperitoneum, limbs and trunk. Majority of cases arise de-novo as primary tumors, while a minority occur as recurrences of well-differentiated liposarcomas. There has been no previous report of dedifferentiated liposarcoma presenting as disseminated intraperitoneal sarcomatosis.

**Presentation of case:**

This report describes a young Asian female with disseminated intraperitoneal sarcomatosis arising from de-novo dedifferentiated liposarcoma, and discusses the diagnostic challenges faced in this highly unusual disease presentation. Initial biopsy was unconclusive, and considerations included sclerosing mesenteritis or an inflammatory myofibroblastic tumor. Fluorescence in situ hybridization was subsequently performed and showed amplification of the MDM2 gene. A diagnosis of dedifferentiated liposarcoma was then made.

**Discussion:**

This was an extremely unusual presentation of advanced dedifferentiated liposarcoma that was eventually diagnosed using fluorescence in situ hybridization. Prognosis is poor and the only viable treatment options was palliative chemotherapy.

**Conclusion:**

The diagnosis of dedifferentiated liposarcoma should be considered early and surgery carried out when still feasible.

## Highlights

• Dedifferentiated liposarcoma can present with disseminated intraperitoneal sarcomatosis.
• Fluorescence in situ hybridization for MDM2 gene amplification is diagnostically discriminative.
• Prognosis is poor and the benefit of chemotherapy remains uncertain.
• Novel targeted therapies involving MDM2 and CKD4 inhibitors may emerge as viable systemic therapy options.

## 1 Introduction

Dedifferentiated liposarcoma is one of the liposarcoma subtypes based on the World Health Organization (WHO) classification of bone and soft tissue tumors [[1], [2], [3]]. It typically displays a high-grade morphology and metastasizes in 15–20% of cases. The most frequent site of dedifferentiated liposarcoma occurrence is in the retroperitoneum, followed by the limbs, trunk, mediastinum, head and neck region and spermatic cord. Most dedifferentiated liposarcomas arise de-novo as primary tumors, with a minority presenting as recurrences of well-differentiated liposarcomas [2]. Reports of liposarcomas arising primarily from intraperitoneal sites such as the omentum or mesentery are rare, with only 40 cases described in the literature [[4], [5], [6], [7], [8], [9], [10], [11], [12], [13], [14], [15], [16], [17], [18], [19], [20], [21], [22], [23], [24], [25], [26]]. Of the 40 cases, 18 were of the dedifferentiated subtype, and all 40 presented with localized resectable disease. This paper describes a case of dedifferentiated liposarcoma with an atypical primary presentation of disseminated intraperitoneal sarcomatosis. To our knowledge, no similar cases have been described in the literature to date. All work has been reported in line with the SCARE criteria [27].

## 2 Presentation of case

The patient is a 37-year-old Asian female with a past surgical history of laparoscopic cholecystectomy for symptomatic cholelithiasis and excision of breast fibroadenomas. She has no other past medical history or significant family history of cancer. She is a smoker of 17 pack-years but does not drink alcohol.

The patient presented with a 1-month history of vague abdominal discomfort and weight loss, and noticed a left abdominal mass 1 week prior to presentation. She denied any early satiety, change in bowel habits, jaundice, melena, or hematochezia. The patient was well nourished, with no signs of anemia. Physical examination revealed a soft abdomen with a palpable left flank mass. This mass was firm, and did not move with respiration. There were no palpable lymph nodes in her supraclavicular fossae, neck and groin.

A computed tomography (CT) scan was arranged, and this showed an intra-peritoneal mass of indeterminate origin in the left flank, with extensive peritoneal and omental nodules. Blood tests revealed that her hemoglobin was 13.6 g/dL (normal range 12.0–16.0 g/dL) and her white cell count was 7.04 × 10^9^/L (normal range 4.0–10.0 × 10^9^/L). Her renal function and liver function were normal. Tumor marker tests were also done, and this showed that her cancer antigen-125 (CA 125) was elevated at 225U/ml (normal range <35.1U/ml) but cancer antigen 19-9 (CA 19-9), carcinoembryonic antigen (CEA) and alpha-fetoprotein (AFP) were within normal limits.

**Fig. 1:**
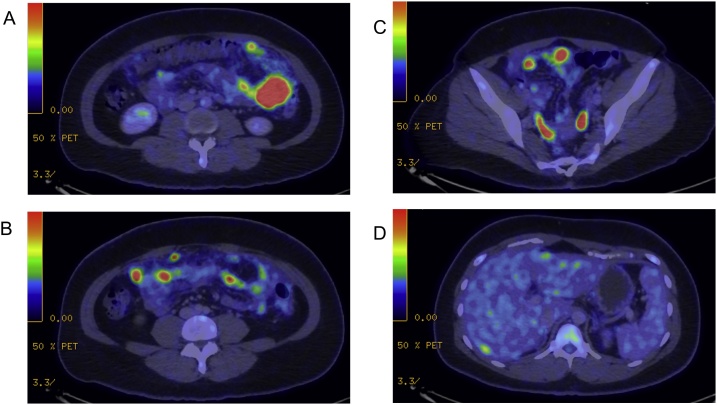
PET-CT scan images showing disseminated FDG-avid foci in A) Omentum, B) Small bowel, C) Pelvis and D) Increased peripheral liver uptake.

She then underwent esophagogastroduodenoscopy and colonoscopy which did not show any intraluminal lesions. A positron emission tomography-computed tomography (PET-CT) scan was performed and confirmed the presence of a fluorodeoxyglucose (FDG)-avid mass in the left flank with extensive omental and peritoneal nodules, but no definite primary was identified (Fig. 1). There was no suspicious FDG uptake in the appendix, colon, stomach or ovaries, nor any distant lesions. These findings were initially suspicious for a primary peritoneal malignancy.

**Fig. 2:**
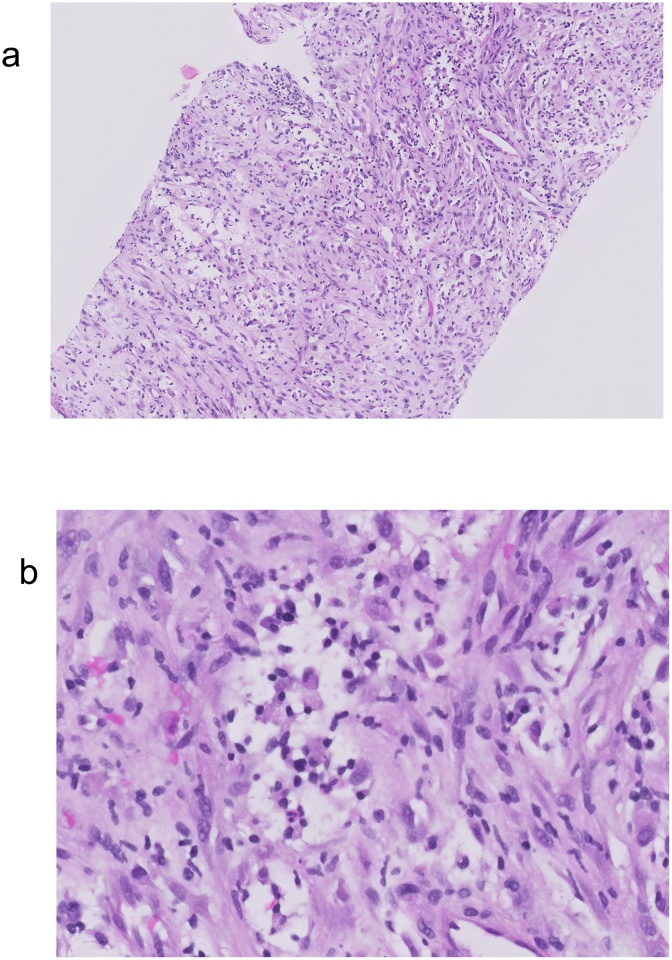
A) Spindle cell proliferation featuring fibroblastic/myofibroblastic-like cells with interspersed inflammatory cells. B) Higher magnification view of the spindle cells showing mild nuclear atypia and interspersed plasma cells and occasional neutrophils.

The patient was referred to a specialized peritoneal malignancy unit, and the case was discussed at the multidisciplinary tumor board meeting. In light of the imaging findings, a recommendation was made for image-guided percutaneous biopsy of the left flank mass. This biopsy showed spindle cells within a loose stroma with mild nuclear atypia and few mitotic figures (Fig. 2A). No necrosis was observed. Interspersed inflammatory cells including plasma cells and neutrophils were noted (Fig. 2B). There was no adipocytic differentiation present. On immunohistochemistry, the lesional cells showed patchy positivity for SMA and CD31. MIB-1 showed a low proliferative index of approximately 2–5%. CAM5.2, DOG-1, CD117, S100, HMB45, CD34, ALK-1, desmin and MSA were negative. β-catenin showed no nuclear reactivity. A few scattered IgG4 positive cells were noted, with very low IgG4+ to IgG+ ratio. No conspicuous acid-fast bacilli were seen on Ziehl–Neelsen stain. A diagnosis of spindle cell lesion with features of fibroblastic or myofibroblastic proliferation was made. Considerations included sclerosing mesenteritis or inflammatory myofibroblastic tumor (IMT).

During the follow up visit, the patient complained of worsening abdominal discomfort and repeat imaging showed enlargement of the peritoneal nodules within a short span of 3 weeks. After discussion with the patient, a decision was made for a diagnostic laparoscopy and surgical resection of the left flank mass for symptomatic relief and additional tissue for a more definitive diagnosis.

**Fig. 3:**
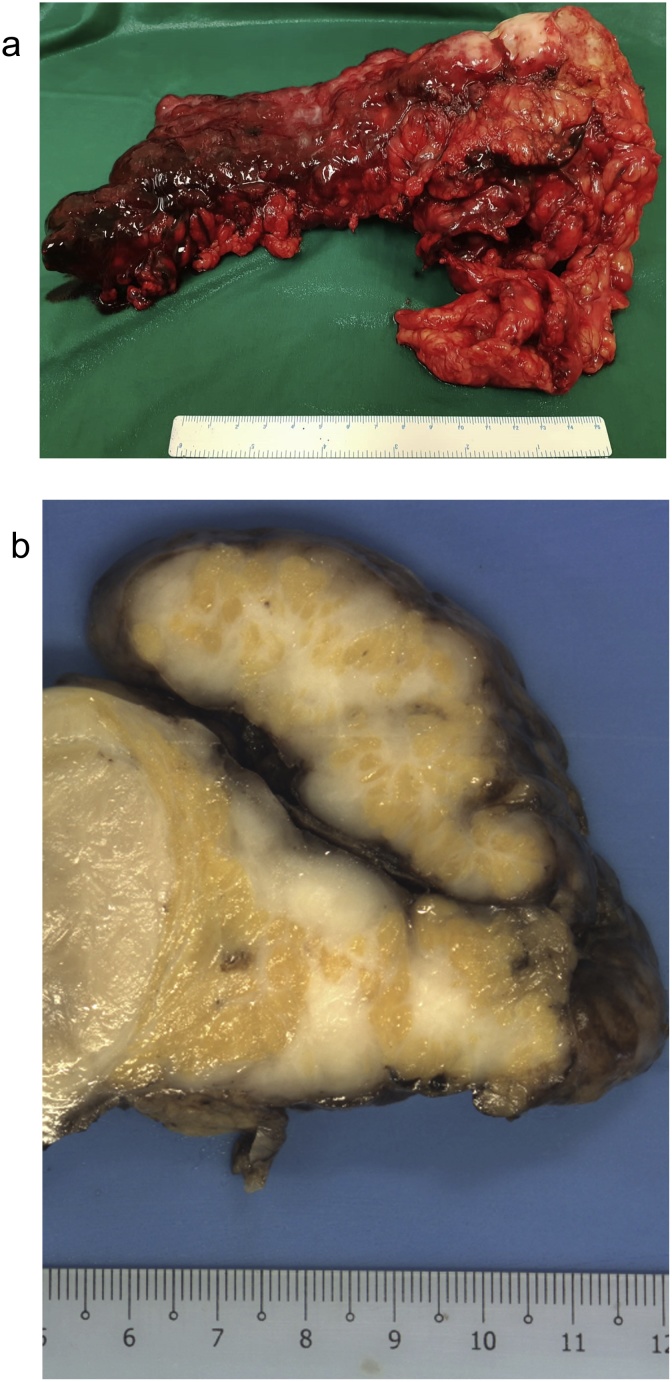
A) Omentectomy specimen containing the dominant tumor nodule. B) Cut surface of the omentum showing whitish, solid tumor with intervening fat.

Intraoperative findings showed extensive peritoneal disease with a peritoneal cancer index (PCI) score of 35 [28]. The culprit lesion was within the greater omentum and extended down into the left flank and pelvis (Fig. 3A). It measured 15 cm and was adherent to the stomach, transverse colon and left abdominal wall. Another large mass measuring 8 cm was found over the mid-jejunum. The appendix, small bowel serosa and mesentery, liver capsule, and splenic capsule were also involved with disease. The initial concern was that of disseminated peritoneal carcinomatosis, but specimens sent for frozen section showed a cellular spindle cell lesion. Omentectomy and appendectomy were performed.

**Fig. 4:**
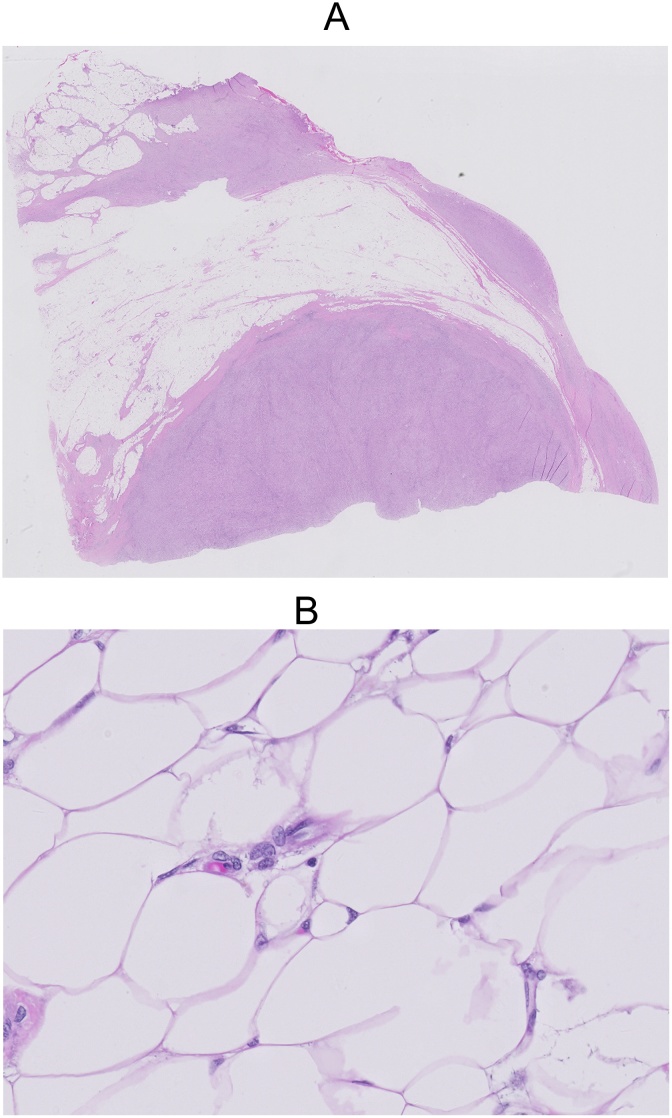
A) Section of the omentum showing solid areas of tumor with an irregular interface with intervening adipose tissue. B) Intervening adipose tissue between the solid tumor areas show cytologic atypia focally.

Formal histopathologic examination of the omental tumor showed a moderately cellular spindle cell proliferation in short fascicles with occasional storiform arrangement. The spindle cell proliferation was admixed with adipose tissue in an irregular, occasionally lace-like pattern (Fig. 4A). The cells showed elongated nuclei with vesicular chromatin, small nucleoli and amphophilic cytoplasm with some cells showing up to a moderate degree of nuclear atypia. There was an associated moderate inflammatory infiltrate which composed of lymphocytes, plasma cells and some eosinophils. The stroma ranged from fibrocollagenous to slightly myxoid. Mitotic figures numbered up to 5 per 10 high power fields. There was no necrosis present. The adipose tissue in between the spindle cell areas showed cytologic atypia focally (Fig. 4B).

Immunohistochemistry was performed and the spindle cells stained positive for SMA, and negative for desmin, caldesmon, CD34, CD21, CD35, ALK, and EBER-ISH. Beta-catenin showed largely cytoplasmic staining. Fluorescence in situ hybridization (FISH) using MDM2/CEP12 probe set was subsequently performed and 88.3% of 60 tumor nuclei scored showed amplification of the MDM2 gene at 12q15. The ratio of MDM2 to CEP12 signals was 5.3. A diagnosis of dedifferentiated liposarcoma (FNCLCC grade 2) was made.

The patient recovered well post-operatively and was discharged on post-operative day 2. Based on the final histology result of a dedifferentiated liposarcoma, she was referred to the medical oncology team and started on doxorubicin chemotherapy.

## 3 Discussion

Dedifferentiated liposarcomas typically occur as discrete tumors in the retroperitoneum, limbs, and trunk. The presentation as disseminated intra-abdominal nodules in this patient was highly unusual and proved to be a diagnostic conundrum. The histologic appearance of proliferating spindle cells with significant admixed inflammatory infiltrates, coupled with an absent adipocytic component in the first biopsy also led to an initial diagnosis of sclerosing mesenteritis or an unusual form of inflammatory myofibroblastic tumor [29].

Dedifferentiated liposarcomas are histologically characterized by a non-lipogenic sarcoma which is often high grade. A well-differentiated component may be present. Amplification of the MDM2 gene is demonstrated in 95% of liposarcomas by FISH [[30], [31], [32]]. This characteristic overexpression of the MDM2 gene opposes p53 function and therefore promotes cell cycle progression [33]. A dense, admixed inflammatory infiltrate can sometimes be observed and such cases may have been mistakenly classified in the past as the inflammatory variant of malignant fibrous histiocytoma, which is now an obsolete entity [2,34]. Occasionally, distinctive whorls of spindle cells can also be observed in the non-lipogenic component of dedifferentiated liposarcomas [[35], [36], [37], [38], [39]]. The unusual presentation of disseminated intraperitoneal disease coupled with the heavy inflammatory infiltrate served as a diagnostic pitfall and the diagnosis of a dedifferentiated liposarcoma was not apparent until the MDM2 FISH was performed.

The cornerstone of treatment for liposarcomas is complete surgical resection, which can be difficult to achieve in abdominal tumors due to their close proximity to vital structures. Given the high local recurrence rates for intra-abdominal and retroperitoneal liposarcomas, radiation therapy can be considered as an adjunct to surgery. The benefit of neoadjuvant radiotherapy in retroperitoneal sarcomas is currently being examined in the STRASS trial [40,41]. Unfortunately, this patient has extensive disseminated intra-peritoneal sarcomatosis which was not amenable to surgical resection.

To date, there have been 4 retrospective single-institution series and 1 phase I study examining the role of cytoreductive surgery (CRS) and intra-peritoneal chemotherapy in the treatment of peritoneal sarcomatosis [[42], [43], [44], [45], [46]]. However, mixed outcomes have been reported and there is marked heterogeneity in histological types, and inclusion and exclusion criteria across the studies. There was also a lack of standardization in the use of hyperthermic intra-peritoneal chemotherapy (HIPEC) or early post-operative intra-peritoneal chemotherapy (EPIC). The Consensus Statement on the Locoregional Treatment of Abdominal Sarcomatosis was published after experts responded to a poll at the 5^th^ International Workshop on Peritoneal Surface Malignancy. It concluded that evidence supporting HIPEC or EPIC for peritoneal sarcomatosis was insufficient. Little has been discussed with respect to systemic therapy options in peritoneal sarcomatosis arising from liposarcomas. The rarity of these presentations has proven to be the greatest barrier to the conduct of formal clinical trials [47].

Response rates to chemotherapy for dedifferentiated liposarcomas has been reported to be ≤12% [48]. Doxorubicin-based chemotherapy has been the standard treatment for soft tissue sarcomas, and ifosfamide also has shown some single-agent activity. Combination therapy with doxorubicin and ifosfamide appears to be the most active regimen, with improved response rates and progression free survival, but no improvement in overall survival. Dacarbazine and a combination of gemcitabine and docetaxel has also been used in advanced soft tissue sarcomas, but little data on efficacy exists for dedifferentiated liposarcomas [40,41]. Our patient was started on palliative single agent doxorubicin chemotherapy, and was counselled of the poor prognosis and response to chemotherapy.

## 4 Conclusion

This case report describes an unusual presentation of disseminated intraperitoneal sarcomatosis and its associated diagnostic challenges. Perhaps earlier diagnosis could have led to earlier commencement of treatment. The benefit of chemotherapy for this patient, however, remains uncertain. Novel targeted therapies involving MDM2 and CKD4 inhibitors are currently being evaluated and may change the landscape of systemic therapy for liposarcomas in the future [3].

## Conflicts of interest

The authors declare that there is no conflict of interest.

## Funding

This research did not receive any specific grant from funding agencies in the public, commercial, or not-for-profit sectors.

## Ethical approval

This case report is exempt from ethical approval in our institution.

## Consent

Written informed consent was obtained from the patient for publication of this case report and accompanying images. A copy of the written consent is available for review by the Editor-in-Chief of this journal on request.

## Author contribution

Cai Mingzhe – Data collection and writing of paper.

Caroline Siew Ching Hsia – Study concept and writing of paper.

Timothy Tay Kwang Yong – Data collection and writing of paper.

Grace Tan Hwei Ching – Study concept, writing of paper and overall supervision.

## Registration of research studies

http://www.researchregistry.com UIN: researchregistry4923

## Guarantor

Grace Tan Hwei Ching.

## Provenance and peer review

Not commissioned, externally peer-reviewed.
